# The American Agriculturist

**Published:** 1881-12

**Authors:** 


*The American Agriculturist.*—We consult the best good of our readers in recommending them to now secure the valuable and important information and most interesting reading matter, including a thousand or more of pleasing and instructive engravings and sketches, that can be obtained at trifling expense in the *American Agriculturist.* This is not merely a farm and garden journal, but is very useful to every housekeeper and to every household in village or country. It has an entertaining and useful department for the little ones. It is a journal that pays to take and read. Try it, and, our word for it, you will not be disappointed. Its constant, persistent exposures of humbugs and swindling schemes are worth far more than the cost of the paper. The 41st annual volume begins January 1st, but those subscribing now for 1882 get the rest of this year free. Terms: $1.50 a year; four copies $5 (English or German edition;) single copy, 15 cts.

N. B.—Those desiring can get an extra or double specimen number post-free for 10 cts., by addressing the Publishers, Orange Judd Co., 751 Broadway, New York.

